# A Novel Thromboplastin-Based Rat Model of Ischemic Stroke

**DOI:** 10.3390/brainsci11111475

**Published:** 2021-11-07

**Authors:** Irina V. Ostrova, Sergei N. Kalabushev, Ivan A. Ryzhkov, Zoya I. Tsokolaeva

**Affiliations:** 1 V.A. Negovsky Research Institute of General Reanimatology, Federal Research and Clinical Center of Intensive Care Medicine and Rehabilitology, 107031 Moscow, Russia; skalabushev@fnkcrr.ru (S.N.K.); iryzhkov@fnkcrr.ru (I.A.R.); tsokolaevazoya@mail.ru (Z.I.T.); 2 Institute of Functional Genomics, Lomonosov Moskow State University, 119991 Moscow, Russia; 3 National Medical Research Center of Cardiology, Russian Ministry of Health, 121552 Moscow, Russia

**Keywords:** embolic stroke, rat model, thromboplastin, brain injury, hippocampus

## Abstract

The thromboembolic ischemia model is one of the most applicable for studying ischemic stroke in humans. The aim of this study was to develop a novel thromboembolic stroke model, allowing, by affordable tools, to reproduce cerebral infarction in rats. In the experimental group, the left common carotid artery, external carotid artery, and pterygopalatine branch of maxillary artery were ligated. A blood clot that was previously formed (during a 20 min period, in a catheter and syringe, by mixing with a thromboplastin solution and CaCl_2_) was injected into the left internal carotid artery. After 10 min, the catheter was removed, and the incision was sutured. The neurological status of the animals was evaluated using a 20-point scale. Histological examination of brain tissue was performed 6, 24, 72 h, and 6 days post-stroke. All groups showed motor and behavioral disturbances 24 h after surgery, which persisted throughout the study period. A histological examination revealed necrotic foci of varying severity in the cortex and subcortical regions of the ipsilateral hemisphere, for all experimental groups. A decrease in the density of hippocampal pyramidal neurons was revealed. Compared with existing models, the proposed ischemic stroke model significantly reduces surgical time, does not require an expensive operating microscope, and consistently reproduces brain infarction in the area of the middle cerebral artery supply.

## 1. Introduction

One of the main requirements for the development of experimental models of cerebrovascular disease is the maximal relevance for real-life clinical practice. The choice of the most appropriate model can be crucial for investigating potential treatment strategies for stroke and its sequelae [1]. Ischemic stroke in humans, in most cases, results from a sudden arterial occlusion (most commonly of the middle cerebral artery) by a thrombus or embolus. Embolic occlusion of the middle cerebral artery (MCAO), with an autologous or heterologous blood clot in rodents, is one of the methods to model focal and multifocal cerebral ischemia, and is highly consistent with the pathogenesis of ischemic stroke in humans [2,3,4]. Changes in the blood flow properties have been studied on thromboembolic models, which should be taken into account when developing both reperfusion and neuroprotective approaches to the treatment of ischemic stroke [5]. The possibility of testing new thrombolytic agents is another important advantage of these models [3,4].

Earlier, in the first experimental model of thromboembolic stroke in rats, proposed by Kudo M. et al. (1982), clots spontaneously formed from arterial blood were used for embolism [6]. The disadvantages of this method include low resistance of the thrombus to the effect of intrinsic fibrinolytic system, high risk of spontaneous recanalization of the thrombus, and, consequently, low reproducibility of brain infarction [7].

Later, a model was developed (Busch E., 1997) using thrombin-induced clots from autologous or allogeneic blood for embolism [8]. Preparation of clots took more than 5 h. The clots obtained in this way are characterized by greater elasticity and a high density of the fibrin network than in spontaneous clots. This is essential for reproducibility of infarct size. At the same time, such clots differ from spontaneously developing clots in a clinical stroke, where thrombin generation depends on the prothrombin plasma level, which varies considerably in different patients. The disadvantages of this method consist of a highly complicated technique of obtaining clots, which makes preparing for the experiment time-consuming; the operation procedure (introduction of a catheter into the internal carotid artery through the external carotid artery) requires expensive equipment, such as an operating microscope in the laboratory. In addition, clots obtained this way are extremely resistant to thrombolytic therapy [7].

Relatively recently, a model of thromboembolic occlusion of the middle cerebral artery in rats was developed, which involved the injection of thrombin directly into the lumen of the MCA, with the resulting clot formation [9,10]. The disadvantages of this method are its invasiveness (craniotomy is required) and poor standardization of clot formation, which reduces the reproducibility of brain infarction, the limitation for assessment of neurological/sensorimotor deficits because of the small size and location of the infarct [7,11].

Thus, the existing models of thromboembolic stroke are technically very complicated and invasive, and the frequency of cerebral hemorrhage and mortality among experimental animals are rather high [12]. A new thrombus formation method might provide the possibility of studying, more precisely, the effect of reperfusion strategies in animal stroke models [1]. The aim of the present study was to develop a clinically relevant model of thromboembolic stroke that allows simulating cerebral infarction in rats using reasonable techniques by researchers.

## 2. Materials and Methods

### 2.1. Animals

The experiments were performed in accordance with protocols approved by the Local Ethics Committee of the Federal Research and Clinical Center of Intensive Care Medicine and Rehabilitology, no. 1/20/3, of 11 March 2020. Animal confinement and experiments were carried out in accordance with accepted national and international bioethical standards, including Directive 2010/63/EU of the European Parliament and of the Council of the European Union on the protection of animals, used for scientific purposes.

A total of 45 adult male Wistar rats (250–400 g) were randomly divided into two groups: the experimental group (n = 40, 322 ± 5 g) and the sham group (control, n = 5, 321 ± 18 g).

### 2.2. Animal Model

**Figure 1:**
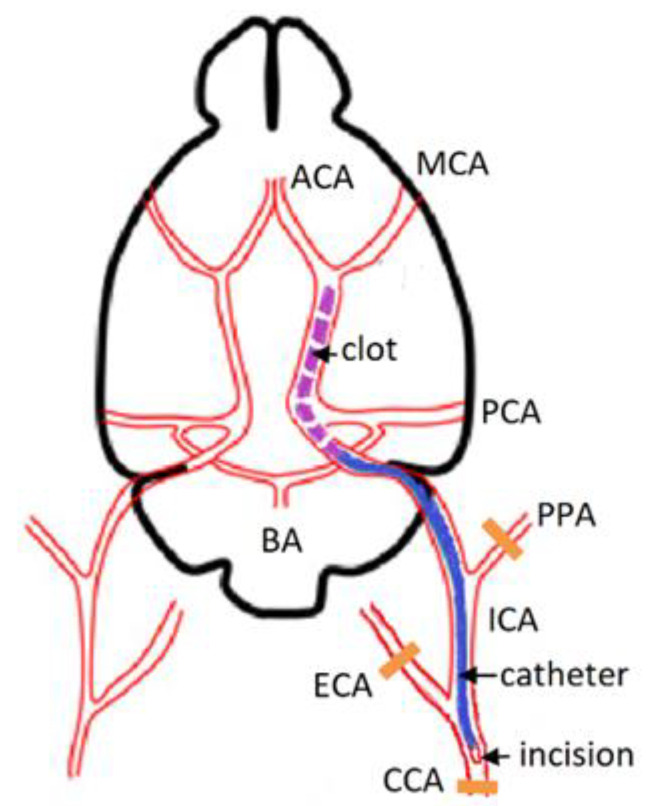
Scheme of thromboembolic occlusion of the MCA. CCA—common carotid artery, MCA—middle cerebral artery, ICA—internal carotid artery, PPA—pterygopalatine artery, BA—basilar artery, ECA—external carotid artery, ACA—anterior cerebral artery, PCA—posterior cerebral artery.

The embolic stroke model was modified from a previous study [13]. Rats were anesthetized with an intraperitoneal injection of 6% chloral hydrate (300 mg/kg) with additional injections of 100 mg/kg dose if the depth of anesthesia was reduced. The skin was cleaned with antiseptic solution. A 2-cm long midline skin incision was made on the anterior surface of the rat’s neck. A 1% lidocaine solution was applied to the incision. The left common carotid artery (CCA) was isolated bluntly and ligated using the silk 4.0 suture. The left external carotid artery (ECA) and internal carotid artery (ICA) were separated close to the skull base. The ICA was carefully separated from the adjacent vagal nerve. The ECA and pterygopalatine artery (PPA) were ligated. Microsurgical scissors were used to make a small incision in the CCA at a 2 mm distal to the bifurcation site. The ready-made composite catheter (with the intravascular part consisting of PU tube, 0.6 × 0.3 × 30 mm, and the extravascular one being a PVC tube, 1.0 × 0.7 × 70 mm, SciCat, Moscow, Russia) was carefully inserted into the ICA through the incision in the CCA at a distance of 14–18 mm from the CCA bifurcation. In the experimental group, 400 µL of arterial blood was collected into a syringe containing 50 µL of thromboplastin solution and 50 µL of 0.025 M CaCl_2_ solution. A standard set of reagents for determination of prothrombin time (Diagem-P, RPA Renam, Moscow, Russia) was used. The syringe was disconnected from the catheter; the latter was flushed with a 0.9% NaCl solution. The blood in the syringe was stirred. After 20 min, the 100-µL clot formed in the syringe was injected through the catheter into the lumen of ICA until reaching the desired position (judged by increased resistance) (Figure 1). In the sham group, the left CCA, ECA, and PPA were ligated and a similar volume (100 µL) of 0.9% NaCl solution was injected through a catheter in the ICA. After 10 min, the catheter was removed, the left CCA was additionally ligated cranially from the puncture site, and the surgical wound was sutured. The animal was transferred to the cage. Paracetamol solution (30 mg/kg intraperitoneally) was administered to relieve pain. The total duration of procedures was 45–60 min.

### 2.3. Physiological Measurements

Before clot injection, ECG, heart rate, respiration rate, and rectal temperature were recorded in the animals using MouseMonitor S system (INDUS Instruments, Webster, TX, USA). Acid-base status and arterial blood gases (pO_2_, pCO_2_, pH, HCO_3_, SO_2_) were analyzed using the CG4+/ CG8+ reagent cartridges for i-STAT analyzer (Abbott Point of Care Inc., Princeton, NJ, USA).

### 2.4. Neurological Deficit Assessment

Twenty-four hours after surgery, neurological status was assessed using a 0–20 point rating scale described by Hunter et al. [14], excluding contralateral reflex (normal score: 20; maximal deficit score: 0). This test evaluated some aspects of neurological functions, including motor, sensory, reflex, and balance functions. For some, animal additional examinations were carried out 2 and 5 days after the surgery.

### 2.5. Histological Evaluation

Histological evaluation: 6 h (n = 4), 24 h (n = 7), 72 h (n = 8), and 6 days (n = 5) after surgery, the animals under deep anesthesia were perfused with a 0.9% NaCl solution and then with a 10% solution buffered formalin. The brain was removed and resuspended in 10% formalin for 48 h and embedded in paraffin according to the standard technique. Frontal brain sections 5–6 µm thick were prepared at the levels of striatum, sensorimotor cortex, and hippocampus (Bregma 1.1 ± 0.5 mm, Bregma −1.1 ± 0.5 mm, Bregma −4.3 ± 0.5 mm, respectively, according to the atlas by Paxinos G. and Watson C., 1986). Histological examination of brain tissue to identify areas of ischemic damage was performed on hematoxylin–eosin stained sections. Images of brain sections were obtained using a ScanScope CS digital scanner (Leica Biosystems, Vista, CA, USA).

The number of viable pyramidal neurons in the CA1 and CA3–4 fields of the left hemisphere hippocampus were counted using 40× objective (Nikon Eclipse Ni-U light microscope, Nikon Corp., Tokyo, Japan) without the examiner knowing the experimental protocol, using a computer-associated image analyzer (NIS-Elements BR software, Nikon Corp., Tokyo, Japan). The cells with a well-defined ellipsoidal or circular nucleus and a clearly distinguishable nucleolus located in the center of the nucleus were categorized as normal viable neurons [15].

### 2.6. Statistical Analysis

The data obtained were processed and evaluated using Statistica 13.0 software package. Differences between groups were analyzed using parametric (one-way ANOVA followed by Tukey’s post hoc test for multiple comparisons for physiological variables) and nonparametric criteria (Mann–Whitney U-criterion for unrelated samples and Wilcoxon W-criterion for related samples) were used and considered statistically significant when *p* < 0.05.

## 3. Results

One-way ANOVA revealed that physiological parameters as well as the body weight measured before surgery did not differ significantly between groups (*p* > 0.05) (Table 1).

The overall mortality rate within the experimental group was 40%. Two rats died during the operation, and 14 of 40 rats died during the first day.

### 3.1. Neurological Status Assessment

**Figure 2:**
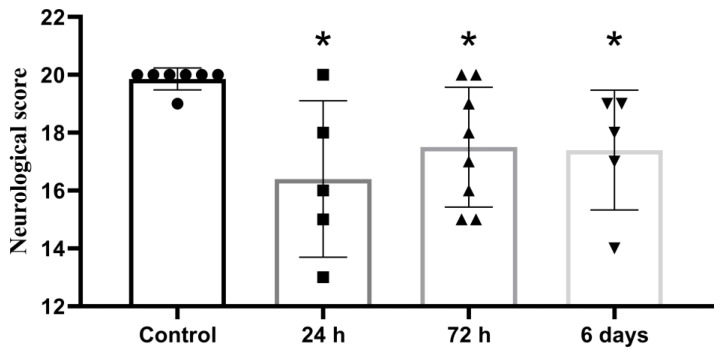
Neurological assessment of animal status 24 h after embolic MCAO; 20-point scale, *—significant difference vs control (*p* ≤ 0.05, Mann-Whitney U-criterion). The data is presented as mean with standard deviation.

A 20-point assessment of neurological status 24 h after surgery revealed impaired motor and behavioral functions in all groups of experimental animals, compared with the control (Figure 2).

**Figure 3:**
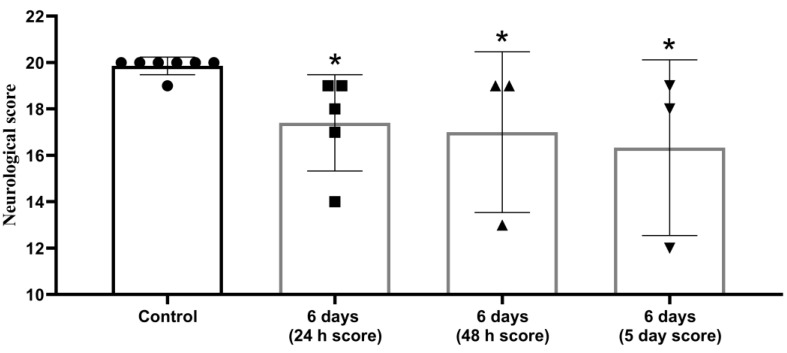
Decrease in neurological status in post stroke period persisted for 5 days after MCAO. The 20-point scale, *—significant difference vs. control (*p* ≤ 0.05. Wilcoxon W-criterion). The data are presented as mean with standard deviation.

Animal status assessment conducted on days 2 and 5, showed persistent neurological abnormalities (Figure 3).

### 3.2. Histology

**Figure 4:**
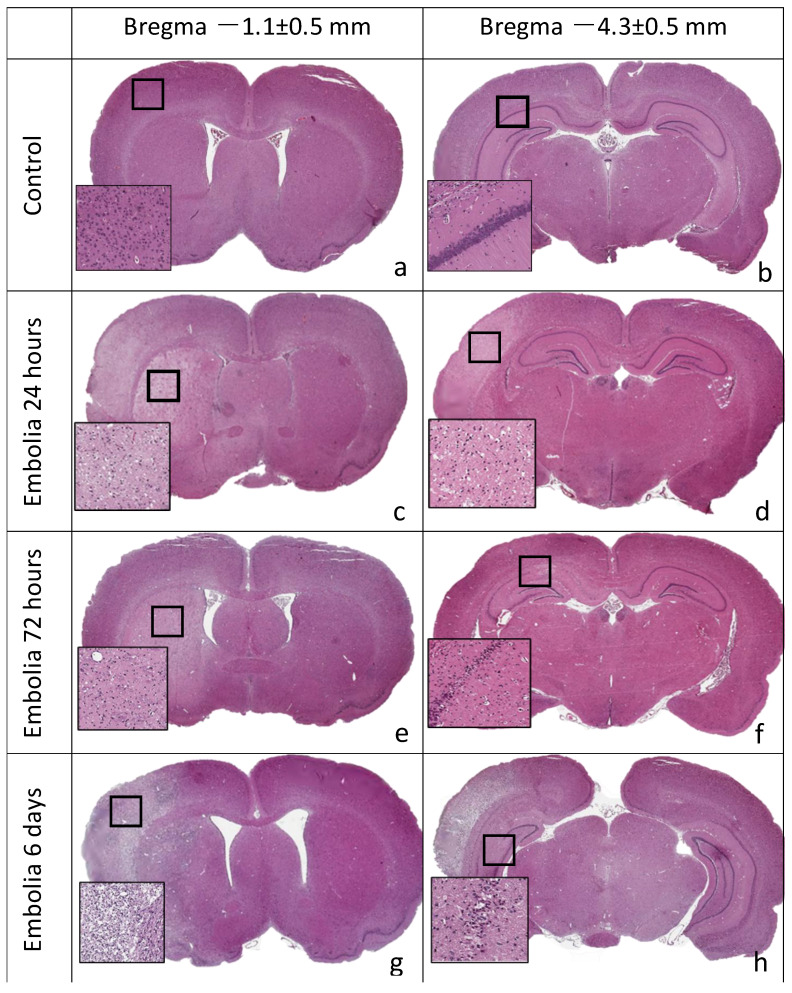
Frontal slices of rat brain, hematoxylin-eosin staining. Ischemic lesions in the territory of the left hemisphere supplied by MCA: striatum, cortex, hippocampus. (**a**,**b**) Sham control, no areas of damage in the ipsilateral and contralateral cerebral hemispheres. (**c**,**d**) A total of 24 h after embolization, infarct areas in the striatum and cortex are pale-stained. (**e**,**f**) A total of 72 h after embolization, infarct areas in the striatum and hippocampus. (**g**,**h**) A total of 6 days after embolization, infarct areas in the striatum, cortex, hippocampus, and thalamus.

Histological examination revealed no areas of brain tissue damage in the sham-operated rats in the ipsilateral or contralateral hemisphere (Figure 4a,b). In 16 of 24 animals (67%) of the experimental group, tissue edema and necrosis were observed in the left middle cerebral artery supply area, i.e., striatum, cortex, and hippocampus. The remaining animals had separate foci of damaged neurons in the cortex and hippocampus. The parietotemporal cortex (seven animals), hippocampus (nine animals), striatum, and thalamic region (nine animals) were most frequently affected. The study of brain sections at different periods after MCAO revealed necrotic foci of varying severity in the cortex and subcortical regions of the ipsilateral hemisphere, after 6 h—in one of four animals, after 24 h—in six of seven rats (Figure 4c,d), after 72 h—in five of eight rats (Figure 4e,f), after 6 days—in four of five rats (Figure 4g,h).

Quantitative analysis of hippocampal pyramidal neuron populations revealed a decrease in the density of normal neurons in the CA1 field by 16.8% (n = 7, *p* < 0.05) 24 h after MCAO, by 26.2% (n = 7, *p* < 0.05) 72 h later, and by 60.5% (n = 5, *p* < 0.05) 6 days later vs. group 2, respectively (Table 2). The CA4 field also shows a decrease in the density of normal neurons by 12.6% (n = 4, *p* < 0.05) after 6 h, by 29.6 (n = 7, *p* < 0.05) after 24 h, by 9.3% (n = 7, *p* ≤ 0.1) after 72 h, and by 19.8% (n = 5, *p* < 0.05) after 6 days vs. the control, respectively (Table 2).

## 4. Discussion

The use of a new modification of the thromboembolic stroke model produced a persistent impairment of motor and behavioral functions in the experimental group animals, as well as necrosis foci in the middle cerebral artery supply areas in the ipsilateral cerebral hemisphere. Quantitative analysis revealed significant damage to the highly hypoxia-sensitive pyramidal neurons of the hippocampus early after MCAO, which persisted throughout the study period. The innovation of the proposed method of modeling thromboembolic stroke (in combination with permanent occlusion of ipsilateral CCA) lies in the use of thromboplastin instead of thrombin. Tissue thromboplastin (tissue factor III) is a phospholipoprotein present in the cell membranes of many tissues. It is the main activator of the external clotting system during secondary (coagulation) hemostasis [16]. During the external pathway of coagulation cascade, the complex of thromboplastin, factor VIIa and Ca^2+^ activates factor X. In its turn, factors Xa, Va, Ca^2+^ and phospholipoproteins (including tissue thromboplastin) form a complex that activates prothrombin and converts it into thrombin [17,18].

Under pathological conditions, tissue thromboplastin can trigger thrombosis formation, both in the arterial and venous systems of the body [19]. In the unstable (ruptured or eroded) atherosclerotic plaque, the massive release of tissue thromboplastin, along with platelet activation and aggregation, is an important pathogenetic factor of atherothrombosis, which is thrombus formation on the surface of an unstable atherosclerotic plaque [18,20,21]. This pathophysiology underlies the atherothrombotic subtype of ischemic stroke. The adhered and aggregated platelets are considered the basis of arterial thrombus due to the high blood flow rate, which determines the clinical significance of antiaggregant therapy in the prevention and treatment of atherothrombotic stroke [22]. Nevertheless, activation of the coagulation cascade, including that under the effect of tissue thromboplastin, leads to fibrin clot formation, which promotes consolidation and growth of a primary loose platelet clot [18,21].

Apparently, tissue thromboplastin and the external coagulation pathway have less pathogenetic significance in the development of cardioembolic ischemic stroke with the underlying atrial fibrillation and pathologies of the heart valve abnormalities [23]. The important factors of thrombin generation and fibrin clot formation in the left atrium are the initial hypercoagulation, reduced blood flow, atrial wall remodeling, and impaired fibrinolysis. The above pathophysiological factors, combined with a long period of thrombus formation, are responsible for the high density of the fibrin clot and the relative resistance of the thromboembolus, causing ischemic stroke to fibrinolytics [24].

There are many approaches to experimental modeling of ischemic stroke in laboratory animals [25]. In some models, thrombin is used as an activator of thrombosis. Blood clots induced by thrombin can be produced in vitro and then injected through a catheter into a cerebral vessel; thus, simulating cardioembolic stroke in humans [8,13]. Alternatively, thrombin can be injected directly into the middle cerebral artery and cause thrombosis in situ [9,10]. Each of the above experimental models has its own advantages, reflecting a particular aspect of the pathogenesis of ischemic stroke in humans. However, most of such animal models are technically cumbersome and, most importantly, do not take into account the phases of blood coagulation preceding thrombin generation (platelet activation and aggregation, coagulation cascade through external and internal pathways), representing only the final (general) pathway of blood coagulation. Moreover, fibrin-rich clots contain a reservoir of enzymatically active thrombin, which is released from clot material during thrombolysis and may have secondary effects, such as enhancement of platelet procoagulant activity, promotion of edema formation, induction of vasospasm, and possibly neurotoxicity [7,26].

Thus, we can specify several advantages and specific features of using thromboplastin to induce thrombosis in the experimental models of ischemic stroke:

1. Despite the formation of a blood clot in vitro, the new thromboplastin-calcium model of ischemic stroke in rats is more feasible than similar models, better reflects the pathogenesis of the atherothrombotic ischemic stroke in humans, and simulates thrombogenesis, starting from the first stages of the external pathway of the coagulation cascade.
2. The high clinical relevance of the novel experimental model is based on taking into account the pathogenesis of perioperative ischemic stroke, which is a major clinical issue, especially in cardiovascular surgery [27].

The issue of clinical relevance of this model for the study of the pathogenesis of cardioembolic ischemic stroke with underlying atrial fibrillation and the creation of appropriate new therapeutic approaches requires further elaboration. In particular, one of the disadvantages of the novel model is the relatively short time of thrombus formation, which, apparently, does not correspond to the real-time dynamics of this process in humans.

In our study, the overall mortality rate within the experimental group was 40%, most of the animals died during the first 24 h after the surgery. Mortality in animal models of ischemic stroke varies widely (1–57%) and is largely determined by animal strain, the invasiveness of surgery, and the extent of the brain lesion [11,25,27]. Large zones of cerebral infarction were detected in the MCA supply area in 67% of survived animals. The remaining animals had heterogeneous foci of neuronal damage in the cortex and subcortical structures. The histological findings were consistent with the impairments in motor and behavioral functions in the same animals 24 h after surgery. Thus, the results of this study (severity and localization of ischemic brain damage) are comparable with the results of similar experimental studies [6,13,27]. However, the relatively high mortality rate in our model can be reduced by improving perioperative laboratory animal care.

In addition, in our model, the clot itself was introduced, not through the external carotid artery, but through the common carotid artery, which is technically much easier to perform and does not require an expensive operating microscope. Thrombus insertion through the common carotid artery was performed in the experiments in the early 1980s [6]. Currently, in thromboembolic stroke modeling, the clot is injected through the external carotid artery in most cases. A modification of thromboembolic MCAO model developed by Zhang RL (1997) is most commonly used in the current studies [28,29]. However, this method requires an expensive operating microscope, which significantly limits its use. On the other hand, introduction of the formed clot through CCA in the absence of an operating microscope reduces the risk of damage to small brain arteries, which results in decreased frequency of cerebral hemorrhage and surgical mortality, and improved reproducibility of cerebral infarcts.

Overall, the advantages of our developed model include:

1. Using thromboplastin instead of thrombin to trigger thrombus formation, which increases the relevance of our model due to greater similarity between the mechanisms of onset and development of ischemic stroke in the rat and in humans.
2. The use of autologous blood clots, which is more relevant than the use of allogeneic material.
3. Reduced cost of the experiment by eliminating the need for expensive equipment, in particular the operating microscope.
4. Obtaining significant structural and functional changes in the brain.

## 5. Conclusions

The proposed novel method of ischemic stroke modeling has a number of advantages over other models, i.e., relative co-effectiveness, ease of performance, relatively low mortality, obtaining significant structural and functional changes in the brain, a fairly good reproducibility and high relevance, which may further contribute to the development of effective techniques for thrombolytic and neuroprotective therapy of ischemic stroke.

## Figures and Tables

**Table 1:** Physiological parameters of the anesthetized rats (prior to MCAO).

Group	Weight, g	pH	РaСО_2_, mm Hg	РaО_2_, mm Hg	НСО_3_	SO_2_	Lactate	HR	RR	Rectal Temperature, °С
Control	321 ± 18	7.34 ± 0.01	43.5 ± 1.1	64.5 ± 4.5	23.6 ± 0.2	90.2 ± 1.8	1.1 ± 0.4	374 ± 27	86 ± 9	35.2 ± 0.5
6 h	339 ± 0.7	7.32 ± 0.01	40.3 ± 3.2	57.6 ± 3.3	20.7 ± 1.2	87.3 ± 1.8	1.4 ± 0.1	380 ± 20	108 ± 11	35.1 ± 0.0
24 h	357 ± 21	7.32 ± 0.01	40.1 ± 0.8	66.0 ± 6.8	20.8 ± 0.8	89.5 ± 3.3	0.9 ± 0.1	367 ± 28	82.7 ± 6	34.8 ± 0.1
72 h	324 ± 6.8	7.31 ± 0.01	42.4 ± 2.9	67.6 ± 4.8	21.1 ± 1.6	90.2 ± 1.9	1.0 ± 0.2	366 ± 23	78.8 ± 6	34.7 ± 0.4
6 days	329 ± 22	7.31 ± 0.01	42.6 ± 4.9	58.6 ± 7.4	21.9 ± 2.9	86 ± 5.0	0.9 ± 0.4	409 ± 18	86.2 ± 8	34.8 ± 0.3

Note: the data are presented as mean ± S.E.M.; HR—heart rate, RR—respiratory rate after the operation. In the sham group, no fatal outcomes were recorded.

**Table 2:** Number of normal viable pyramidal neurons in fields CA1 and CA4 of the hippocampus at different periods after MCAO, per 1 mm of cell layer length.

Hippocampal Field	Control	6 h	24 h	72 h	6 days
CA1	206.5 (199.1; 209.5)	202.5 (97.3; 216.8)	171.9 * (133.1; 188.5)	152.5 * (64.9; 178.7)	81.5 * (13.0; 124.6)
CA4	118.4 (118.4; 119.5)	103.5 * (87.5; 110.5)	83,3 * (71.7; 103.5)	107.4 ^#^ (63.4; 123.2)	94.9 * (84.8; 98.9)

Note. Data presented as medians and quartiles. *—*p* < 0.05, ^#^—*p* ≤ 0.1 vs. the controls, Mann–Whitney U-test.

## Data Availability

Not applicable.
